# Racial/Ethnic Disparities in Mortality: Contributions and Variations by Rurality in the United States, 2012–2015

**DOI:** 10.3390/ijerph16030436

**Published:** 2019-02-02

**Authors:** Jeffrey E. Hall, Ramal Moonesinghe, Karen Bouye, Ana Penman-Aguilar

**Affiliations:** Centers for Disease Control and Prevention, Office of Minority Health and Health Equity, 4770 Buford Highway, TW-3, Atlanta, GA 30341, USA; dzu4@cdc.gov (J.E.H.); keh2@cdc.gov (K.B.); bpv4@cdc.gov (A.P.-A.)

**Keywords:** health disparities, rurality, race/ethnicity, place

## Abstract

The value of disaggregating non-metropolitan and metropolitan area deaths in illustrating place-based health effects is evident. However, how place interacts with characteristics such as race/ethnicity has been less firmly established. This study compared socioeconomic characteristics and age-adjusted mortality rates by race/ethnicity in six rurality designations and assessed the contributions of mortality rate disparities between non-Hispanic blacks (NHBs) and non-Hispanic whites (NHWs) in each designation to national disparities. Compared to NHWs, age-adjusted mortality rates for: (1) NHBs were higher for all causes (combined), heart disease, malignant neoplasms, and cerebrovascular disease; (2) American Indian and Alaska Natives were significantly higher for all causes in rural areas; (3) Asian Pacific islanders and Hispanics were either lower or not significantly different in all areas for all causes combined and all leading causes of death examined. The largest contribution to the U.S. disparity in mortality rates between NHBs and NHWs originated from large central metropolitan areas. Place-based variations in mortality rates and disparities may reflect resource, and access inequities that are often greater and have greater health consequences for some racial/ethnic populations than others. Tailored, systems level actions may help eliminate mortality disparities existing at intersections between race/ethnicity and place.

## 1. Introduction

In the United States, place of residence plays an important role in shaping population health. Mortality and morbidity vary greatly across the geographic continuum between urban and rural and for populations residing in metropolitan versus non-metropolitan areas [1,2]. Our place of residence influences not only how we live and our health trajectories but also from what we are likely to die [3,4,5,6,7]. In particular, in the U.S., age-adjusted mortality rates and percentages of potentially excess deaths from the five leading causes of death are higher for non-metropolitan versus metropolitan areas [8]. This includes deaths from what some refer to as “diseases of despair”—suicide and drug use [9,10,11].

The noted differences in mortality rates and excess deaths among non-metropolitan and metropolitan communities are geographically influenced by social determinants of health. The places where populations reside differ by demographic, economic, and social characteristics [8]. Social correlates and determinants of health associated with morbidity and mortality in non-metropolitan areas and rural areas in particular, include: socioeconomic conditions (e.g., poverty levels [12,13]; demographic characteristics (e.g., population age structure [14]); and systemic and access factors (e.g., workforce shortages and service supply inadequacies, facility and service closures [1,13,15,16]). Moreover, those residing in non-metropolitan areas have poorer mental health; are more likely to smoke, and exhibit obesity; and less likely to be physically active, use seat belts, and possess adequate health insurance [2,17,18] as compared to those residing in metropolitan areas. Such factors and circumstances may increase risks for death.

The value of disaggregating non-metropolitan and metropolitan area deaths in illustrating place-based health effects has been demonstrated [8]. However, whether and how place interacts with social characteristics such as race and ethnicity has been less firmly established, making it important to examine subpopulation differences in distributions of outcome (such as mortality from the leading causes of death). Increasing understanding of the interrelations between place and variables such as race/ethnicity is important because the health related effects of place might not operate the same for all populations. The health statuses, sociodemographic characteristics, and social position of distinct racial/ethnic populations living in the same places may differ vastly and diverge considerably from those of their counterparts living in other areas [19]. Such differences could amplify or attenuate both racial/ethnic and place-based health disparities, vary the contributions to national level disparities in mortality associated with causes of death, and call for different public health actions to enable optimal health to be accessed regardless of one’s residence or race/ethnicity. 

This paper completes three actions to generate insights regarding patterns of place-related health disparities. We first characterize racial/ethnic variations in mortality associated with the five leading causes of death and in sociodemographic characteristics by rural designation. Next we compare age adjusted mortality rates for non-Hispanic black, American Indian/Alaska Native (AI/AN), Asian/Pacific Islander, and Hispanic populations to those of non-Hispanic white populations in each of 6 rurality designations constituting metropolitan and non-metropolitan areas by calculating absolute disparities. Lastly, we assess the contributions of absolute disparities in mortality rates between non-Hispanic black and non-Hispanic populations residing in different rurality designations to national absolute disparities in age-specific mortality rates in the United States. The methodologies and findings reported may further strengthen rationales for disaggregating geographically organized data; provide insights about variations in health and health disparities existing at intersections of place, age, and race/ethnicity; and promote refinement of methods by which health disparities in the U.S. are defined, understood, and addressed.

## 2. Methods

To study the sociodemographic characteristics and the mortality rates by rurality, we used the 2013 NCHS urban-rural classification scheme for U.S. counties [19]. This classification scheme categorizes counties as large central metropolitan (R1), large fringe metropolitan (R2), medium metropolitan (R3), small metropolitan (R4), micropolitan (R5), and noncore or rural (R6). R1 includes counties in metropolitan statistical areas (MSAs) of at least a population of one million that contain the entire population of the largest principal city of the MSA, have their entire population contained in the largest principal city of the MSA, or contain at least 250,000 inhabitants of any principal city of the MSA. R2 includes counties in MSAs of 1 million or more population that did not qualify as large central metro counties. R3 includes counties in MSAs of populations of 250,000 to 999,999. R4 includes counties in MSAs of populations less than 250,000. R5 includes counties in micropolitan statistical areas, and R6 includes non-metropolitan counties that did not qualify as micropolitan. The metro areas in the United States consist of R1, R2, R3, and R4, and non-metro areas consist of R5 and R6. Of the 3143 U.S. counties identified in 2013, a total of 68, 868, 373, 358, 641, and 1325 counties were in R1, R2, R3, R4, R5, and R6 areas respectively and 30.5%, 24.7%, 20.9%, 9.2%, 8.7%, and 6.1% of the U.S. population respectively lived in these areas [19]. 

To estimate the sociodemographic characteristics by race and ethnicity in R1, R2, R3, R4, R5, and R6 areas, we used the self-reported data from the combined 2012–2015 annual surveys of the Behavioral Risk Factor Surveillance System (BRFSS). BRFSS is an annual state-based, random-digit–dialed telephone (landline and cell telephone) survey of the noninstitutionalized U.S. population aged ≥18 years. The median weighted survey response rate for all states and DC in 2012–2015 ranged from 45.2% to 47.2%. Detailed information about the BRFSS survey and sample design are available elsewhere [20]. We obtained a data set that identified county FIP codes in the 2012–2015 BRFSS through a data use agreement and merged it with the BRFSS data to identify the urban-rural classification of the counties. We used the following sociodemographic measures for sociodemographic characteristics: race/ethnicity, age (18–44, 45–64, and ≥65 years), sex, educational attainment (less than high school, high school diploma or General Education Development [GED] certificate, some college, or college graduate), marital status (married or not married), household income (<$25,000, $25,000–$49,999, $50,000–$74,999, or ≥$75,000), employment status (employed or not employed), and U.S. census region (Northeast, Midwest, South, or West) [21]. Categories for race/ethnicity included non- Hispanic white; non-Hispanic black; and a combined Asian and Native Hawaiian/other Pacific Islander (“Asian and NHOPI”) category. The survey data were analyzed using the DESCRIPT procedures in SAS-callable SUDAAN, version 11.0.1 (Research Triangle Institute, Research Triangle Park, NC, USA) that takes into account the complex survey design of the BRFSS, and the sample weights were adjusted for combining four years of BRFSS data. Statistical significance of comparisons between non-Hispanic whites and other population groups was determined with t-test at *α* = 0.05.

We analyzed mortality data using the Web based tool CDC WONDER [22]. CDC WONDER provides data based on all resident death certificates filed in the 50 states and District of Columbia. We obtained age adjusted mortality rates for non-Hispanic black, non-Hispanic white, American Indian or Alaska Native (non-Hispanic), Asian and Pacific Islander (non-Hispanic), and Hispanic populations for all-cause mortality and for the 5 leading causes of death in the United States: heart disease, malignant neoplasms, chronic lower respiratory disease, cerebrovascular disease, and unintentional injury during 2012–2015. We calculated absolute disparities in age adjusted mortality rates between non-Hispanic white and other race/ethnicity groups using non-Hispanic white as the referent group. Statistical significance of disparities was determined with Z tests at *α* = 0.05.

Next, we developed a method to assess the contribution of deaths between two population groups in each area, *R_i_*, *i* = 1, 2, 3, 4, 5, 6, to the absolute disparities in age-specific mortality rates in the U.S. between the two population groups. Let MR_A_ and MR_B_ be the mortality rates for two population subgroups, *A* and *B*, and *MR_Ai_* and *MR_Bi_* be the mortality rates for the two population groups in each area, *R_i_*, *i* = 1, 2, 3, 4, 5, 6. Then *MR_A_* − *MR_B_* can be expressed as:

(1) $$MR_{A}-MR_{B}=\sum_{i=1}^{6}(w_{Ai}MR_{Ai}-w_{Bi}MR_{Bi})$$

where *w_Ai_* and *w_Bi_* are the proportions of the U.S. populations of subgroups *A* and *B* residing in *R_i_* (e.g., if, hypothetically, 100,000 members of subgroup *A* reside in *R_i_* and there are 1,000,000 members of subgroup A in the U.S., then *w_A_*_1_ = 0.1) The contribution of deaths between the two population groups A and B for the area *R_i_*, *i* = 1, 2, 3, 4, 5, 6, is given by $w_{Ai}MR_{Ai}-w_{Bi}MR_{Bi}$. Note that $w_{Ai}MR_{Ai}-w_{Bi}MR_{Bi}$ could be negative if the number of deaths in *R_i_* divided by the U.S. population size for group A is lower than that of group B. We used this method to assess the contribution of deaths between non-Hispanic blacks and non-Hispanic whites in each area, *R_i_*, for age-specific mortality rates in the U.S. for all-causes and the five leading causes of deaths for age groups 20–44, 45–64, and ≥65.

## 3. Results

The BRFSS study sample for most variables contained 1,767,768 adult respondents in the 50 states and DC. The sample included the following: 1,431,493 non-Hispanic white (80.98%), 148,560 non-Hispanic black (8.40%), 28,193 AI/AN (1.59%), 120,454 Hispanic (6.81%), and 39,068 Asian and NHOPI (2.21%).

Table 1, Table 2, Table 3, Table 4, Table 5 and Table 6 present social demographic characteristics of adult residents in R1, R2, R3, R4, R5, and R6 areas respectively. Compared with non-Hispanic whites, members of racial/ethnic minority populations tended to be younger in all the areas considered except for AIAN in R5. The residents in metropolitan areas (R1, R2, R3, and R4) tended to be younger than the residents in non-metropolitan areas (R5 and R6). For example, the population aged 18–44 years ranged 39.7% to 43.0% in non-metropolitan areas and 45.4% to 50.7% in metropolitan areas. There were higher percentages of non-Hispanic black females in all the areas except in R3, whereas higher percentages of Hispanic males were in all the areas except in R1 compared with respective non-Hispanic white subpopulations. Higher percentages of non-Hispanic black (62.3% to 71.3%), Hispanic (51.0% to 56.8%), and AI/ANs (54.3% to 62.6 %) were not married compared to non-Hispanic whites (41.0% to 48.4%) in each area. There was considerable variation by rurality designation in the distribution of racial/ethnic minority populations across Census regions. Almost 93.9%, 88.1%, 73.9%, 68.3% and 62.6% of the non-Hispanic black population in R6, R5, R4, R3, and R2 areas respectively, but only 39.4% in R1, lived in the southern region. Around 41%, 40.6%, 42.1%, and 59.1% of Hispanics in R2, R3, R5, and R6 areas respectively lived in the south and 45.1%, 44.0%, and 46.5% of Hispanics in R1, R3, and R4 areas respectively lived in the west. Of AI/AN in R2 and R3, around 45% lived in the South and 43.1% in R4 lived in the west. Of Asians and NHOPIs in R1, R3, and R5, 59.2%, 57.0%, and 50.8% respectively lived in the west.

All the racial/ethnic minority groups except for Asians and NHOPI (combined) tended to have lower levels of educational attainment in all the areas compared to respective non-Hispanic white populations. Higher percentages of Hispanics had less than a high school diploma in all the six areas with 40.4%, 33.8%, 38.4%, 38.2%, 40.8%, and 44.2% having less than high school diploma in R1, R2, R3, R4, R5, and R6 respectively compared to non-Hispanic whites with 5.9%, 7.4%, 9.1%, 11.0%, 12.9%, and 15.3%, respectively in corresponding areas. On the other hand, Asians and NHOPI had higher percentages of college graduates (52.5%, 57.4%, 41.8%, 41.2%, 33.9%, and 35.4% in R1, R2, R3, R4, R5, and R6, respectively) compared to corresponding non-Hispanic white populations (39.2%, 32.8%, 27.7%, 23.9%, 19.0%, and 16.0%, respectively). The unemployment percentages for AI/ANs were higher (ranged from 48.3% to 55%) compared to non-Hispanic whites (ranged from 41.7% to 47.7%) in all the areas. Similarly, unemployment percentages for non-Hispanic blacks (ranged from 47.4% to 53.7%) were higher compared to non-Hispanic whites in all the areas except in R2. However, Hispanics, Asians, and NHOPI had either lower or not significantly different unemployment percentages in all the areas compared to non-Hispanic whites. 

Table 7 gives the age-adjusted mortality rates for non-Hispanic white, non-Hispanic black, AIAN, Hispanic and Asian/Pacific Islander (API) populations for all causes and for the five leading causes of death: heart disease, malignant neoplasms, chronic lower respiratory disease, cardiovascular disease, and unintentional injury during 2012–2015 in R1, R2, R3, R4, R5, and R6 areas. For non-Hispanic whites, the age adjusted mortality rates for all causes and the five leading causes of death were higher in non-metropolitan areas compared to metropolitan areas (All comparisons of individual non-metro areas to individual metro areas were significant). For example, the age adjusted mortality rate for all causes for non-Hispanic whites ranged from 705.8 to 777.8 per 100,000 population in metropolitan areas whereas the same rate for non-metropolitan areas ranged from 826.5 to 842.9 per 100,000 population.

Table 8 provides the absolute disparities in age-adjusted mortality rates for non-Hispanic black, AIAN, Hispanic and API populations compared to non-Hispanic white population in these areas. The age adjusted mortality rates for non-Hispanic blacks were higher compared to non-Hispanic whites for all causes, heart disease, malignant neoplasms, and cerebrovascular disease, but lower for chronic lower respiratory disease and unintentional injury in all the areas. The highest disparity in age adjusted mortality rates for all causes between non-Hispanic blacks and non-Hispanic whites was in R1 (181.7 per 100,000 population) and the lowest was in R2 (64.4 per 100,000 population). The age adjusted mortality rate for AIAN for all causes was higher compared to non-Hispanic whites (with a disparity of 190.4 per 100,000 population) in R6, but significantly lower in R2 (with a disparity of −164.3 per 100,000 population). The age adjusted mortality rates for AI/AN were either lower or had no significant difference compared to the age adjusted mortality rates for non-Hispanic whites in all the areas for heart disease, malignant neoplasms, chronic lower respiratory disease, and cerebrovascular disease, whereas they were higher for unintended injury in all areas except R2. The age-adjusted mortality rates for all causes and the five leading causes of deaths for both APIs and Hispanics were either lower or had no significant difference compared to the age adjusted mortality rates for non-Hispanic whites in all the areas. The largest disparity in age adjusted mortality rates for all causes between APIs and non-Hispanic whites was in R6 (−465.9 per 100,000 population). Similarly, the largest disparity in age adjusted mortality rates for all causes between Hispanics and non-Hispanic whites was also in R6 (−272.4 per 100,000 population).

Table 9 gives the mortality rates for all causes of death for the non-Hispanic black and non-Hispanic white populations in R1, R2, R3, R4, R5, R6, and in the U.S. for the age groups 20–44, 45–64, and ≥65 during 2012–2015, and the contribution of deaths in the two populations in each area to the disparity in mortality rates between the two populations in the U.S. The mortality rates for the non-Hispanic white and non-Hispanic black populations for all causes for the age group 20–44 in the U.S. were 135.63 and 188.89 per 100,000 population respectively, which resulted in an absolute disparity of 53.26 per 100,000 population between the two populations in the U.S. The contributions from R1, R2, and R3 tended to increase the disparity, whereas the contributions from R4, R5, and R6, tended to decrease the disparity between the two populations. The largest contribution to the disparity in the U.S. was in R1 (56.61). The reason for this is the large proportion of non-Hispanic black population in R1 (42%). The mortality rates for non-Hispanic blacks and non-Hispanic whites for all causes for the age group ≥65 were 4435.3 and 4353.1 per 100,000 population in the U.S. resulting in a disparity of −82.2 between the two population groups. The contribution of deaths from all the areas except from R1 tended to decrease the disparity in mortality rates in the U.S. between the two populations. The disparity in mortality rates between non-Hispanic black and non-Hispanic white populations in R1 for this age group was −58.2 (4381.7–4439.9) per 100,000 population in R1. However, the contribution of this difference in death rates between the two populations in R1 per 100,000 U.S. population was 1000.51. This higher contribution to the disparity in mortality rates from R1 resulted in a small disparity of −82.2 per 100,000 in mortality rates in the U.S. between the two population groups, despite larger disparities in some areas (e.g., R2 where the disparity was −466.4 (3835.2–4301.6). 

## 4. Discussion

We identified variability in mortality for the five leading causes of death and all-cause mortality in the U.S. by rurality and race/ethnicity, when we considered their intersection. For example, looking across all rurality designations and all racial/ethnic populations, heart disease mortality ranged from 77.2 per 100,000 population among APIs in non-fringe metro areas to 247.5 per 100,000 population among non-Hispanic blacks in non-core areas. For malignant neoplasms, the range was from 86 per 100,000 population among APIs in non-core areas to 208.2 per 100,000 population for non-Hispanic blacks in micropolitan areas. However, the data were nevertheless patterned by the individual dimensions of race/ethnicity and rurality, with, for example, non-Hispanic blacks faring consistently worse (regardless of rurality) than the referent group of non-Hispanic whites for three of the five causes (heart disease, malignant neoplasms, and cerebrovascular disease) and Hispanics and APIs faring consistently better than non-Hispanic whites across all five causes. These findings align with cause specific findings reported in the limited comparable literature stratifying mortality data by both race/ethnicity and rurality [23,24]. 

An example of a pattern by rurality broadly consistent with other recent studies is that non-metropolitan areas (micropolitan and non-core) tended to fare worse than metropolitan ones [1,8,13,25,26,27,28]. When we considered the contribution of rurality to age-specific disparities in all-cause mortality between non- Hispanic blacks and non-Hispanic whites, it was not surprising that, due to the high proportion of non-Hispanic blacks residing there in combination with the elevated black-white disparities occurring there, large central metropolitan areas were the greatest drivers of national disparities. Notably, however, the contributions of rural designation were not uniform across age categories. 

Our findings suggest that historical patterns of residence determine where health disparities concentrate nationally, whereas place of residence may influence the amounts and kinds of racial/ethnic health disparities observed sub-nationally by rurality. Waves of migration concentrated non-Hispanic blacks in urban areas during the late 1800s and early 1900s [29,30]. While this concentration has decreased over time, large proportions of the non-Hispanic black population continue to live in urban areas with insufficient material, physical, and economic resources. These include low-income families unable to relocate as low-skilled, high paying jobs vanished with the shift to a service based economy and as residential segregation practices limited residential mobility [29,31]. The latter set of influences situated non-Hispanic Blacks in locations within Metropolitan areas separated from those occupied by non-Hispanic whites. Differences in the past and present conditions of the neighborhoods in which different families were historically concentrated may partially explain our finding regarding the contribution of large central metro areas to the national disparity in all-cause mortality for non-Hispanic black and non-Hispanic white populations. 

Place-based variations in mortality rates, health status, and health disparities may reflect differences in opportunity structures (e.g., proportion of jobs where workers are likely to remain in poverty despite being employed; equity of access to high quality jobs with adequate benefits and pay), economic patterns (e.g., job growth; unemployment/underemployment), and access factors (e.g., health care access; availability of employer provided health insurance) [16,32,33,34]. Such contextual differences create resource, capital, and institutional access inequities that have greater consequences for health for some racial/ethnic populations than others [16,32,33,34]. This creates situations where mortality rates for the same racial/ethnic population and their positioning relative to those of other populations can differ vastly depending on geography.

Differences in contextual factors may also explain variations in compositional factors linked to mortality and disparity differences. Contextual influences such as migration and job growth patterns influence which populations locate and remain in specific areas and their chances to satisfy their needs. Therefore, the varying proportions of persons of different race/ethnicities with household incomes below the Federal Poverty Threshold, some college education or a college degree, or a regular source of health care reported here and elsewhere may arise from place-based differences in economic, educational, or health opportunities [32,35]. 

Examinations of health disparities involving different levels of rurality often occur at the national level, with rural areas often to urban areas without regard to the full continuum of rurality. One element frequently ignored is that, beyond purely geographic disparities, different areas may show different racial/ethnic disparities and provide substantially different contributions to national disparities. In the present analysis, we considered a fuller continuum of rurality and compared racial and ethnic populations.

It is has been noted that urban areas should be a focus of much interest because the largest proportion of the U.S. population resides in urban areas. Yet, to our knowledge, this assumption has not considered in light of patterns of health disparities by rurality. The methodology articulated in this paper provides a means to quantify differences in the contributions of different geographic locales to overall patterns of health disparities for the nation. In this first use of this methodology, using all-cause mortality as an example, the largest contribution to national level disparities between non-Hispanic whites and non-Hispanic blacks (considered on the absolute scale) was, in fact, provided by large central metropolitan areas. Our analysis also shows that, while urban areas consistently are a location of notable divergences in mortality between non-Hispanic whites and non-Hispanic blacks, these divergences occur across all levels of geography. Our results suggest that the greatest gains in decreasing black-white disparities in health at the national level may be obtainable by addressing health determinants responsible for between group variations in the health of those residing in large central metropolitan areas. However, this does not obviate the need to address social determinants responsible for disparities in other places. The specific social factors to which disparities may be attributed are likely to differ across the continuum of rurality.

## 5. Limitations and Practical Implications

The findings in this report are subject to several limitations. First, although the Office of Management and Budget data standards separate categories for NHOPIs and Asians [36] these groups were combined here because of small numbers of decedents in some geographies. Collapsing categories in this manner allowed data for these decedents to be used but did not allow characterization of the unique and relative circumstances of NHOPIs and Asians individually. Second, the combined category for NHOPIs and Asians may represent something different in our BRFSS findings versus our mortality findings from CDC Wonder. Third, adjustments for possible racial and ethnic misclassifications, which have been shown to be a key consideration for some racial and ethnic populations (e.g., Hispanics and American Indians [37,38]), were not attempted. 

All-cause mortality was used for demonstrative purposes—to illustrate the usefulness of our methodology for assessing the comparative contribution (by geographic area) of deaths within two population groups to absolute disparities in age-specific mortality rates observed at the national level. However, the presented methodology can be extended in several ways. For example, it could be used to examine patterns of disparity contributions for specific causes of death and, potentially, for key sources of morbidity. Analyses of other specific causes of morbidity and mortality could reveal differing contribution patterns for various combinations of age, race/ethnicity, and rurality. For the sake of simplicity, we limited our geographically structured analyses to mortality rates for all causes of death for only the non-Hispanic black and non-Hispanic white populations. However, the methodology can be extended to other racial/ethnic populations and to populations demarcated by other socially significant characteristics (e.g., socioeconomic status, gender, sexual orientation, etc.). 

Lastly, the methodology can be applied to other elements of relevant geographic hierarchies (e.g., counties, states, regions, etc.). While the current paper examined disparities in mortality for the U.S. as a nation, it may be particularly informative to examine disparities by rurality and race/ethnicity (considered jointly) for more granular geographic entities such as regions, divisions, or states (where feasible and appropriate). Such analyses could suggest new directions for efforts to more fruitfully and specifically address the varying role of place in the social patterning of health and health disparities.

## 6. Conclusions

This report documents and extends epidemiologic descriptions of mortality disparities existing at intersections between place and race/ethnicity. This opens the door for work to identify and address the unique and shared factors underlying these differences and disparities. Specific attention to the interplay of social and individual determinants of health over time, as health is shaped by the structures and social ecologies of different places is warranted. Such research could identify place-specific conditions influencing how race/ethnicity is experienced and operates in expanding or limiting health opportunities. Moreover, characterizing social processes creating different population health trajectories is essential to address the effects of forces for which rural and urban categories and sub-categories may be proxies (e.g., state and local political, economic, and health infrastructures, policies, and practices; neighborhood, school, and workplace conditions; risk exposure levels etc.). This in turn, could suggest interventions to alter context specific factors driving racial/ethnic disparities in the leading causes of death that are more potent and relevant than current practice. 

## Figures and Tables

**Table 1:** Sociodemographic characteristics of rural dwelling adults * by race and ethnicity: Behavioral Risk Factor Surveillance System, United States, 2012–2015.

Sociodemographic Characteristic	Black, Non-Hispanic % (95% CI)	White, Non-Hispanic % (95% CI)	Hispanic % (95% CI)	Asian or NHOPI, Non-Hispanic % (95% CI)	AIAN, Non-Hispanic % (95% CI)	Total
Age (years)						
18–44	43.7 ^†^ (42.0, 45.4)	36.9 (36.5, 37.4)	66.0 ^†^ (63.9, 68.1)	60.5 ^†^ (51.4, 68.9)	49.2 ^†^ (46.9, 51.6)	39.7 (39.2, 40.1)
45–64	38.9 (37.4, 40.4)	37.4 (37.0,37.8)	25.3 ^†^ (23.5, 27.3)	32.0 (23.5, 41.9)	37.0 (34.8, 39.3)	36.7 (36.3, 37.1)
65+	17.4 ^†^ (16.4, 18.4)	25.7 (25.4, 25.7)	8.6 ^†^ (7.6, 9.7)	7.5 ^†^ (5.3, 10.6)	13.7 ^†^ (12.4, 15.1)	23.6 (23.3, 23.9)
Sex						
Male	45.9 ^†^ (44.3, 47.6)	48.4 (48.0, 48.9)	54.7 ^†^ (52.4, 57.0)	50.2 (41.8, 58.5)	48.8 (46.4, 51.2)	48.6 (48.2, 49.0)
Female	54.1 ^†^ (52.4, 55.7)	51.6 (51.1, 52.0)	45.3 ^†^ (43.0, 47.6)	49.8 (41.5, 58.2)	51.2 (48.8, 53.6)	51.4 (50.9, 51.8)
Marital status						
Not married	68.1 ^†^ (66.6, 69.5)	41.0 (40.5, 41.4)	51.0 ^†^ (48.6, 53.4)	43.8 (35.9, 52.0)	61.1 ^†^ (58.9, 63.4)	44.3 (43.9, 44.7)
Married	31.9 ^†^ (30.5, 33.4)	59.0 (58.6, 59.5)	49.0 ^†^ (46.6, 51.4)	56.2 (48.0, 64.0)	38.8 ^†^ (36.6, 41.1)	55.7 (55.2, 56.1)
Educational attainment						
<High School	28.3 ^†^ (26.8, 29.9)	15.3 (14.9, 15.7)	44.2 ^†^ (41.8, 46.6)		25.9 (23.7, 28.3) ^†^	18.3 (18.0, 18.7)
High School diploma/GED	40.1 ^†^ (38.5, 41.7)	37.7 (37.3, 38.1)	31.4 ^†^ (29.3, 33.6)	29.1 ^†^ (22.3, 37.0)	35.9 (33.7, 38.2)	37.4 (37.0, 37.8)
Some college	23.1 ^†^ (21.8, 24.5)	31.0 (30.6, 31.4)	18.2 ^†^ (16.5, 20.0)	22.7 ^†^ (17.3, 29.2)	29.6 (27.5, 31.9)	29.5 (29.1, 29.8)
College graduate	8.4 ^†^ (7.8, 9.1)	16.0 (15.8, 16.3)	6.2 ^†^ (5.4, 7.1)	35.4 ^†^ (28.2, 43.3)	8.5 ^†^ (7.5, 9.6)	14.8 (14.5, 15.0)
Annual household income						
<$25,000	61.8 ^†^ (60.1, 63.6)	31.8 (31.3, 32.2)	53.1 ^†^ (50.5, 55.7)	28.6 (21.6, 36.7)	56.3 ^†^ (53.7, 58.9)	36.1 (35.7, 36.5)
$25,000–$49,999	25.2 ^†^ (23.7, 26.8)	30.7 (30.2, 31.1)	27.7 ^†^ (25.5, 30.0)	25.6 (18.7, 34.0)	25.1 ^†^ (23.0, 27.4)	29.9 (29.5, 30.3)
$50,000–$74,999	7.4 ^†^ (6.5, 8.4)	16.8 (16.5, 17.2)	10.6 ^†^ (9.0, 12.5)		9.6 ^†^ (8.0, 11.5)	15.5 (15.2, 15.8)
≥$75,000	5.5 ^†^ (4.9, 6.3)	20.7 (20.3, 21.1)	8.6 ^†^ (7.2, 10.2)	28.5 (20.6, 38.0)	8.9 ^†^ (7.8, 10.3)	18.5 (18.2, 18.9)
Employment status						
Not employed	53.7 ^†^ (52.1, 55.4)	47.7 (47.2, 48.1)	38.9 ^†^ (36.6, 41.2)	34.8 ^†^ (27.9, 42.3)	55.0 ^†^ (52.6, 57.4)	47.8 (47.3, 48.2)
Employed	46.3 ^†^ (44.6, 47.9)	52.3 (51.9, 52.7)	61.1 ^†^ (58.8, 63.4)	65.2 ^†^ (57.7, 72.1)	45.0 ^†^ (42.6, 47.4)	52.2 (51.8, 52.6)
Census Region						
Northeast	1.2 ^†^ (0.8, 1.8)	9.3 (9.1, 9.6)	3.1 ^†^ (2.3, 4.3)	8.8 (5.5, 13.9)	4.3 ^†^ (3.2, 5.8)	8.1 (7.9, 8.4)
Midwest	4.0 ^†^ (3.3, 4.8)	36.6 (36.2, 37.0)	14.9 ^†^ (13.5, 16.4)	25.5 ^†^ (20.1, 31.8)	24.9 ^†^ (23.1, 26.9)	32.3 (31.9, 32.6)
South	93.9 ^†^ (92.7, 94.8)	43.9 (43.5, 44.3)	59.1 ^†^ (56.8, 61.3)	37.0 (29.6, 45.2)	32.5 ^†^ (30.3, 34.9)	48.6 (48.2, 49.0)
West	1.0 ^†^ (0.6, 1.7)	10.1 (9.9, 10.4)	22.9 ^†^ (21.2, 24.7)	28.6 ^†^ (20.3, 38.7)	38.2 ^†^ (36.0, 40.5)	11.0 (10.7, 11.2)

Abbreviations: CI = Confidence Interval. NHOPI = Native Hawaiian or other Pacific Islander. AIAN = American Indian/Alaska Native. * Adults were defined as persons aged ≥18 years. ^†^ *t*-test *p* < 0.05 for significant difference between non-Hispanic white respondents and respondents in another racial/ethnic category. Estimates not reported due to relative standard error >30%.

**Table 2:** Sociodemographic characteristics of micropolitan dwelling adults * by race and ethnicity: Behavioral Risk Factor Surveillance System, United States, 2012–2015.

Sociodemographic Characteristic	Black, Non-Hispanic % (95% CI)	White, Non-Hispanic % (95% CI)	Hispanic % (95% CI)	Asian or NHOPI, Non-Hispanic % (95% CI)	AIAN, Non-Hispanic % (95% CI)	Total
Age (years)						
18–44	50.0 ^†^ (48.4, 51.5)	39.7 (39.3, 40.2)	66.5 ^†^ (64.9, 68.0)	58.7 ^†^ (54.3, 63.0)	47.1 ^†^ (44.6, 49.6)	43.0 (42.6, 43.4)
45–64	35.6 (34.2, 37.0)	36.3 (36.0, 36.7)	25.1 ^†^ (23.7, 26.4)	27.3 ^†^ (23.6, 31.4)	38.0 (35.6, 40.4)	35.3 (35.0, 35.6)
65+	14.4 ^†^ (13.6, 15.3)	23.9 (23.7, 24.2)	8.5 ^†^ (7.7, 9.3)	13.9 ^†^ (11.1, 17.3)	14.9 ^†^ (13.4, 16.6)	21.7 (21.5, 22.0)
Sex						
Male	46.2 ^†^ (44.6, 47.8)	48.8 (48.4, 49.2)	52.5 ^†^ (50.8, 54.2)	46.8 (42.4, 51.2)	49.1 (46.6, 51.6)	48.9 (48.5, 49.3)
Female	53.8 ^†^ (52.2, 55.4)	51.2 (50.8, 51.6)	47.5 ^†^ (45.8, 49.2)	53.2 (48.8, 57.6)	50.9 (48.4, 53.4)	51.1 (50.7, 51.5)
Marital status						
Not married	67.9 ^†^ (66.6, 69.5)	42.6 (42.2, 43.0)	51.6 ^†^ (49.8, 53.3)	48.8 ^†^ (35.9, 52.0)	60.5 ^†^ (58.9, 63.4)	45.6 (45.2, 446.0)
Married	32.1 ^†^ (30.6, 33.5)	57.4 (57.0, 57.8)	48.4 ^†^ (46.7, 50.2)	51.2 ^†^ (46.8, 55.6)	39.5 ^†^ (37.1, 41.9)	54.4 (54.0, 54.8)
Educational attainment						
<High School	24.4 ^†^ (23.0, 25.9)	12.9 (12.6, 13.2)	40.8 ^†^ (39.0, 42.6)	8.6 ^†^ (6.2, 11.8)	24.7 ^†^ (22.4, 27.2)	16.1 (15.8, 16.5)
High School diploma/GED	36.6 (35.1, 38.1)	35.6 (35.2, 36.0)	30.0 ^†^ (28.5, 31.5)	26.8 ^†^ (22.9, 31.0)	36.6 (34.2, 38.9)	35.2 (34.8, 35.5)
Some college	28.6 ^†^ (27.2, 30.1)	32.5 (32.1, 32.9)	21.9 ^†^ (20.6, 23.4)	30.7 (26.9, 34.9)	29.5 ^†^ (27.3, 31.9)	31.3 (30.9, 31.6)
College graduate	10.4 ^†^ (9.8, 11.2)	19.0 (18.7, 19.2)	7.3 ^†^ (6.7, 8.0)	33.9 ^†^ (29.9, 38.1)	9.1 ^†^ (8.2, 10.2)	17.4 (17.2, 17.7)
Annual household income						
<$25,000	59.6 ^†^ (57.9, 61.3)	29.3 (28.9, 29.7)	51.8 ^†^ (50.0, 53.7)	31.1 (27.2, 35.3)	53.4 ^†^ (50.7, 56.0)	33.7 (33.4, 34.1)
$25,000–$49,999	23.9 ^†^ (22.5, 25.3)	28.9 (28.5, 29.3)	28.6 (26.9, 30.3)	31.0 (26.1, 36.3)	25.6 ^†^ (23.4, 27.9)	28.5 (28.1, 28.9)
$50,000–$74,999	8.3 ^†^ (7.3, 9.4)	17.8 (17.5, 18.1)	10.2 ^†^ (9.0, 11.5)	13.3 ^†^ (10.8, 16.2)	10.3 ^†^ (8.7, 12.1)	16.3 (16.0, 16.6)
≥$75,000	8.2 ^†^ (7.3, 9.2)	24.0 (23.6, 24.3)	9.4 ^†^ (8.4, 10.5)	24.6 (20.7, 29.0)	10.8 ^†^ (9.2, 12.7)	21.5 (21.1, 21.8)
Employment status						
Not employed	52.8 ^†^ (51.2, 54.4)	46.4 (46.0, 46.8)	40.8 ^†^ (39.1, 42.5)	43.7 (39.3, 48.3)	51.4 ^†^ (48.9, 53.9)	46.5 (46.1, 46.8)
Employed	47.2 ^†^ (45.6, 48.8)	53.6 (53.2, 54.0)	59.2 ^†^ (57.5, 60.9)	56.3 (51.7, 60.7)	48.6 ^†^ (46.1, 51.1)	53.5 (53.2, 53.9)
Census Region						
Northeast	2.3 ^†^ (1.8, 3.0)	14.4 (14.1, 14.7)	4.4 ^†^ (3.6, 5.5)	8.0 ^†^ (5.8, 10.9)	5.1 ^†^ (4.0, 6.5)	12.5 (12.2, 12.8)
Midwest	7.5 ^†^ (6.7, 8.4)	36.5 (36.1, 36.8)	17.7 ^†^ (16.6, 18.9)	19.9 ^†^ (16.9, 23.3)	17.3 ^†^ (15.4, 19.3)	32.3 (32.0, 32.6)
South	88.1 ^†^ (86.9, 89.2)	34.1 (33.7, 34.5)	42.1 ^†^ (40.3, 43.9)	21.3 ^†^ (17.9, 25.0)	39.7 ^†^ (37.3, 42.3)	38.7 (38.3, 39.0)
West	2.1 ^†^ (1.6, 2.8)	15.0 (14.7, 15.2)	35.8 ^†^ (34.3, 37.3)	50.8 ^†^ (46.4, 55.2)	37.9 ^†^ (35.7, 40.2)	16.6 (16.3, 16.8)

Abbreviations: CI = Confidence Interval. NHOPI = Native Hawaiian or other Pacific Islander. AIAN = American Indian/Alaska Native. * Adults were defined as persons aged ≥18 years. ^†^ *t*-test *p* < 0.05 for significant difference between non-Hispanic white respondents and respondents in another racial/ethnic category. Estimates not reported due to relative standard error >30%.

**Table 3:** Sociodemographic characteristics of small metropolitan dwelling adults * by race and ethnicity: Behavioral Risk Factor Surveillance System, United States, 2012–2015.

Sociodemographic Characteristic	Black, Non-Hispanic % (95% CI)	White, Non-Hispanic % (95% CI)	Hispanic % (95% CI)	Asian or NHOPI, Non-Hispanic % (95% CI)	AIAN, Non-Hispanic % (95% CI)	Total
Age (years)						
18-44	52.9 ^†^ (51.4, 54.5)	41.5 (41.1, 41.9)	66.6 ^†^ (65.1, 68.1)	69.5 ^†^ (66.4, 72.4)	51.5 ^†^ (48.4, 54.5)	45.6 (45.2, 46.0)
45-64	33.5 ^†^ (32.2, 34.9)	35.0 (34.6, 35.4)	25.3 ^†^ (24.0, 26.7)	21.3 ^†^ (18.8, 24.0)	35.9 (33.1, 38.9)	33.7 (33.3, 34.0)
65+	13.5 ^†^ (12.7, 14.4)	23.5 (23.2, 23.8)	8.1 ^†^ (7.4, 9.0)	9.2 ^†^ (7.6, 11.1)	12.6 ^†^ (11.0, 14.4)	20.7 (20.5, 21.0)
Sex						
Male	47.8 (46.2, 49.3)	48.7 (48.3, 49.1)	50.6 ^†^ (48.9, 52.3)	49.0 (45.5, 52.5)	52.3 ^†^ (49.2, 55.4)	48.9 (48.5, 49.3)
Female	52.2 (50.7, 53.8)	51.3 (50.9, 51.7)	49.4^†^ (47.7, 51.1)	51.0 (47.5, 54.5)	47.7 (44.6, 50.8)	51.1 (50.7, 51.5)
Marital status						
Not married	68.1 ^†^ (66.7, 69.5)	44.1 (43.7, 44.5)	52.3 ^†^ (50.7, 54.0)	50.0 ^†^ (46.5, 53.6)	62.6 ^†^ (59.6, 65.6)	47.3 (46.9, 47.7)
Married	31.9 ^†^ (30.5, 33.3)	55.9 (55.5, 56.3)	47.7 ^†^ (46.0, 49.3)	50.0 ^†^ (46.4, 53.5)	37.4 ^†^ (34.4, 40.4)	52.7 (52.3, 53.1)
Educational attainment						
<High School	19.1 ^†^ (17.8, 20.4)	11.0 (10.7, 11.4)	38.2 ^†^ (36.5, 39.9)	6.5 ^†^ (4.8, 8.9)	23.8 ^†^ (20.9, 26.9)	14.4 (14.1, 14.8)
High School diploma/GED	36.3 ^†^ (34.8, 37.9)	31.9 (31.5, 32.3)	28.2 ^†^ (26.8, 29.6)	20.5 ^†^ (17.8, 23.4)	35.8 ^†^ (32.9, 38.8)	31.8 (31.4, 32.2)
Some college	31.4 ^†^ (29.9, 32.8)	33.2 (32.8, 33.6)	24.8 ^†^ (23.3, 26.3)	31.8 (28.3, 35.4)	30.1 ^†^ (27.4, 33.0)	32.2 (31.8, 32.5)
College graduate	13.2 ^†^ (12.4, 14.1)	23.9 (23.6, 24.2)	8.8 ^†^ (8.2, 9.5)	41.2 ^†^ (37.9, 44.6)	10.3 ^†^ (9.0, 11.7)	21.6 (21.4, 21.9)
Annual household income						
<$25,000	52.1 ^†^ (50.4, 53.8)	26.9 (26.5, 27.3)	51.6 ^†^ (49.8, 53.5)	29.6 (26.2, 33.3)	49.9 ^†^ (46.7, 53.2)	31.8 (31.4, 32.3)
$25,000–$49,999	26.2 (24.7, 27.7)	27.5 (27.1, 27.9)	27.0 (25.4, 28.7)	28.1 (24.7, 31.8)	28.2 (25.2, 31.4)	27.3 (26.9, 27.7)
$50,000–$74,999	9.9 ^†^ (8.9, 11.0)	17.8 (17.4, 18.1)	9.3 ^†^ (8.3, 10.4)	13.2 ^†^ (10.8, 16.0)	9.9 ^†^ (8.2, 11.8)	16.1 (15.8, 16.4)
≥$75,000	11.8 ^†^ (10.7, 13.0)	27.9 (27.5, 28.3)	12.0 ^†^ (10.9, 13.2)	29.1 (25.9, 32.5)	12.0 ^†^ (10.1, 14.2)	24.7 (24.4, 25.1)
Employment status						
Not employed	48.1 ^†^ (46.5, 49.6)	45.8 (45.4, 46.3)	41.3 ^†^ (39.7, 43.0)	45.0 (41.4, 48.5)	53.4 ^†^ (50.3, 56.5)	45.7 (45.3, 46.1)
Employed	51.9 ^†^ (50.4, 53.5)	54.2 (53.7, 54.6)	58.7 ^†^ (57.0, 60.3)	55.0 (51.5, 58.6)	46.6 ^†^ (43.5, 49.7)	54.3 (53.9, 54.7)
Census Region						
Northeast	4.7 ^†^ (4.0, 5.4)	12.8 (12.5, 13.1)	6.4 ^†^ (5.6, 7.3)	8.0 ^†^ (6.4, 10.0)	6.1 ^†^ (4.8, 7.6)	11.3 (11.0, 11.5)
Midwest	18.7 ^†^ (17.4, 20.1)	31.7 (31.4, 32.1)	13.1 ^†^ (12.1, 14.2)	32.6 (29.4, 35.9)	21.3 ^†^ (19.0, 23.8)	28.7 (28.4, 29.0)
South	73.9 ^†^ (72.4, 75.3)	37.0 (36.6, 37.4)	34.0 ^†^ (32.3, 35.7)	26.0 ^†^ (22.9, 29.3)	29.6 ^†^ (26.6, 32.7)	39.7 (39.3, 40.0)
West	2.8 ^†^ (2.2, 3.4)	18.5 (18.2, 18.7)	46.5 ^†^ (44.8, 48.1)	33.5 ^†^ (30.2, 36.9)	43.1 ^†^ (40.1, 46.1)	20.4 (20.1, 20.7)

Abbreviations: CI = Confidence Interval. NHOPI = Native Hawaiian or other Pacific Islander. AIAN = American Indian/Alaska Native. *Adults were defined as persons aged ≥18 years. ^†^ *t*-test *p* < 0.05 for significant difference between non-Hispanic white respondents and respondents in another racial/ethnic category. Estimates not reported due to relative standard error >30%.

**Table 4:** Sociodemographic characteristics of medium metropolitan dwelling adults * by race and ethnicity: Behavioral Risk Factor Surveillance System, United States, 2012–2015.

Sociodemographic Characteristic	Black, Non-Hispanic % (95% CI)	White, Non-Hispanic % (95% CI)	Hispanic % (95% CI)	Asian or NHOPI, Non-Hispanic % (95% CI)	AIAN, Non-Hispanic % (95% CI)	Total
Age (years)						
18–44	54.4 ^†^ (53.4, 55.3)	40.5 (40.2, 40.8)	65.4 ^†^ (64.5, 66.3)	58.7 ^†^ (56.6, 80.8)	50.6 ^†^ (48.1, 53.2)	46.5 (46.2, 46.8)
45–64	32.7 ^†^ (31.9, 33.6)	36.2 (35.9, 36.5)	26.3 ^†^ (25.5, 27.1)	28.5 ^†^ (26.7, 30.4)	34.5 (32.2, 36.9)	34.0 (33.7, 34.3)
65+	12.9 ^†^ (12.4, 13.4)	23.3 (23.1, 23.5)	8.3 ^†^ (7.9, 8.7)	12.8 ^†^ (11.4, 14.3)	14.8 ^†^ (13.3, 16.5)	19.5 (19.3, 19.7)
Sex						
Male	46.7 ^†^ (45.7, 47.7)	48.3 (48.0, 48.6)	50.7 ^†^ (49.7, 51.6)	50.4 (48.3, 52.5)	49.4 (46.8, 51.9)	48.6 (48.3, 48.9)
Female	53.3 ^†^ (52.3, 54.3)	51.7 (51.4, 52.0)	49.3 ^†^ (48.4, 50.3)	49.6 (47.5, 51.7)	50.6 (48.1, 53.2)	51.4 (51.1, 51.7)
Marital status						
Not married	69.1 ^†^ (68.2, 69.9)	43.8 (43.5, 44.1)	54.3 ^†^ (53.3, 55.3)	44.8 (42.7, 46.9)	58.2 ^†^ (55.6, 60.7)	48.2 (47.9, 48.5)
Married	30.9 ^†^ (30.1, 31.8)	56.2 (55.9, 56.5)	45.7 ^†^ (44.7, 46.7)	55.2 (53.1, 57.3)	41.8 ^†^ (39.3, 44.4)	51.8 (51.5, 52.1)
Educational attainment						
<High School	17.9 ^†^ (17.0, 18.8)	9.1 (8.9, 9.3)	38.4 ^†^ (37.4, 39.4)	7.2 ^†^ (6.0, 8.7)	21.7 ^†^ (19.3, 24.3)	14.6 (14.3, 14.8)
High School diploma/GED	33.5 ^†^ (32.6, 34.4)	29.5 (29.2, 29.8)	27.4 ^†^ (26.6, 28.3)	21.5 ^†^ (19.8, 23.3)	31.5 (29.2, 33.9)	29.3 (29.1, 29.6)
Some college	32.3 ^†^ (31.4, 33.2)	33.7 (33.4, 34.0)	24.5 ^†^ (23.7, 25.4)	29.5 ^†^ (27.4, 31.6)	33.3 (30.9, 35.7)	32.0 (31.7, 32.3)
College graduate	16.3 ^†^ (15.8, 16.9)	27.7 (27.4, 27.9)	9.6 ^†^ (9.2, 10.1)	41.8 ^†^ (39.8, 43.8)	13.5 ^†^ (12.1, 15.0)	24.1 (23.9, 24.3)
Annual household income						
<$25,000	48.6 ^†^ (47.6, 49.7)	23.4 (23.1, 23.7)	52.7 ^†^ (51.6, 53.8)	23.1 (21.2, 25.0)	46.1 ^†^ (43.4, 48.9)	30.7 (30.4, 31.0)
$25,000–$49,999	26.4 (25.5, 27.3)	25.7 (25.4, 26.0)	26.0 (25.1, 27.0)	22.7 ^†^ (20.9, 24.7)	27.7 (25.1, 30.3)	25.7 (25.5, 26.0)
$50,000–$74,999	11.3 ^†^ (10.7, 12.0)	17.4 (17.2, 17.7)	9.0 ^†^ (8.4, 9.7)	16.0 (14.3, 17.8)	10.5 ^†^ (9.1, 12.1)	15.4 (15.2, 15.6)
≥$75,000	13.7 ^†^ (13.0, 14.4)	33.5 (33.2, 33.8)	12.3 ^†^ (11.6, 13.0)	38.2 ^†^ (35.9, 40.5)	15.7 ^†^ (13.9, 17.6)	28.2 (27.9, 28.4)
Employment status						
Not employed	47.4 ^†^ (46.4, 48.3)	45.0 (44.7, 45.3)	42.8 ^†^ (41.8, 43.8)	42.1 ^†^ (40.0, 44.3)	53.0 ^†^ (50.4, 55.5)	44.9 (44.6, 45.2)
Employed	52.6 ^†^ (51.7, 53.6)	55.0 (54.7, 55.3)	57.2 ^†^ (56.2, 58.2)	57.9 ^†^ (55.7, 60.0)	47.0 ^†^ (44.5, 49.6)	55.1 (54.8, 55.4)
Census Region						
Northeast	10.5 ^†^ (9.9, 11.1)	19.4 (19.2, 19.6)	9.8 ^†^ (9.3, 10.3)	14.3 ^†^ (13.0, 15.6)	10.5 ^†^ (8.8, 12.4)	16.7 (16.6, 16.9)
Midwest	14.2 ^†^ (13.5, 14.9)	20.3 (20.1, 20.6)	5.6 ^†^ (5.2, 6.0)	10.4 ^†^ (9.4, 11.5)	12.9 ^†^ (11.3, 14.7)	17.0 (16.8, 17.2)
South	68.3 ^†^ (67.3, 69.2)	39.9 (39.6, 40.2)	40.6 (39.7, 41.6)	18.4 ^†^ (16.9, 19.9)	44.6 ^†^ (42.1, 47.2)	42.3 (42.0, 42.6)
West	7.0 ^†^ (6.5, 7.7)	20.3 (20.1, 20.6)	44.0 ^†^ (43.0, 45.0)	57.0 ^†^ (55.0, 59.0)	32.0 ^†^ (29.7, 34.4)	24.0 (23.7, 24.2)

Abbreviations: CI = Confidence Interval. NHOPI = Native Hawaiian or other Pacific Islander. AIAN = American Indian/Alaska Native. *Adults were defined as persons aged ≥18 years. ^†^ *t*-test *p* < 0.05 for significant difference between non-Hispanic white respondents and respondents in another racial/ethnic category. Estimates not reported due to relative standard error >30%.

**Table 5:** Sociodemographic characteristics of large fringe metropolitan dwelling adults * by race and ethnicity: Behavioral Risk Factor Surveillance System, United States, 2012–2015.

Sociodemographic Characteristic	Black, Non-Hispanic % (95% CI)	White, Non-Hispanic % (95% CI)	Hispanic % (95% CI)	Asian or NHOPI, Non-Hispanic % (95% CI)	AIAN, Non-Hispanic % (95% CI)	Total
Age (years)						
18–44	52.5 ^†^ (51.5, 53.5)	39.4 (39.0, 39.7)	66.1 ^†^ (65.1, 67.1)	63.3 ^†^ (61.6, 65.0)	41.8 (38.1, 45.6)	45.4 (45.1, 45.7)
45–64	34.7 ^†^ (33.8, 35.6)	38.4 (38.1, 38.7)	26.7 ^†^ (25.7, 27.6)	28.3 ^†^ (26.7, 29.9)	42.2 ^†^ (38.7, 45.8)	36.0 (35.7, 36.3)
65+	12.8 ^†^ (12.2, 13.4)	22.3 (22.0, 22.5)	7.2 ^†^ (6.8, 7.8)	8.4 ^†^ (7.5, 9.4)	16.0 ^†^ (13.9, 18.5)	18.6 (18.4, 18.8)
Sex						
Male	46.5 ^†^ (45.4, 47.5)	48.1 (47.7, 48.4)	49.2 ^†^ (48.1, 50.4)	49.7 (47.9, 51.4)	50.7 (47.0, 54.4)	48.1 (47.8, 48.4)
Female	53.5 ^†^ (52.5, 54.6)	51.9 (51.6, 52.3)	50.8 ^†^ (49.6, 51.9)	50.3 (48.6, 52.1)	49.3 (45.6, 53.0)	51.9 (51.6, 52.2)
Marital status						
Not married	62.3 ^†^ (61.3, 63.3)	41.4 (41.0, 41.7)	53.8 ^†^ (52.7, 55.0)	36.4 ^†^ (34.6, 38.1)	54.3 ^†^ (50.6, 57.9)	45.1 (44.8, 45.4)
Married	37.7 ^†^ (36.7, 38.7)	58.6 (58.3, 59.0)	46.2 ^†^ (45.0, 47.3)	63.6 ^†^ (61.9, 65.4)	45.7 ^†^ (42.1, 49.4)	54.9 (54.6, 55.2)
Educational attainment						
<High School	12.4 ^†^ (11.6, 13.2)	7.4 (7.2, 7.6)	33.8 ^†^ (32.6, 34.9)	4.9 ^†^ (3.9, 6.1)	17.9 ^†^ (15.2, 21.0)	11.1 (10.9, 11.4)
High School diploma/GED	29.5 ^†^ (28.6, 30.5)	27.7 (27.4, 28.0)	27.1 (26.1, 28.1)	15.1 ^†^ (13.7, 16.7)	33.5 ^†^ (29.9, 37.3)	27.2 (26.9, 27.5)
Some college	33.5 ^†^ (32.5, 34.5)	32.0 (31.7, 32.4)	24.7 ^†^ (23.7, 25.7)	22.6 ^†^ (21.0, 24.2)	32.7 (29.4, 36.2)	30.8 (30.5, 31.1)
College graduate	24.6 ^†^ (23.8, 25.4)	32.8 (32.5, 33.1)	14.4 ^†^ (13.8, 15.1)	57.4 ^†^ (55.6, 59.2)	15.9 ^†^ (13.8, 18.1)	30.8 (30.6, 31.1)
Annual household income						
<$25,000	32.3 ^†^ (31.3, 33.4)	17.5 (17.2, 17.7)	44.9 ^†^ (43.7, 46.2)	16.1 (14.6, 17.7)	37.2 ^†^ (33.6, 41.0)	22.6 (22.3, 22.8)
$25,000–$49,999	26.5 ^†^ (25.5, 27.5)	21.3 (21.0, 21.5)	26.1 ^†^ (25.0, 27.2)	17.5 ^†^ (16.1, 19.1)	25.7 ^†^ (22.3, 29.4)	22.3 (22.0, 22.6)
$50,000–$74,999	14.7 ^†^ (13.9, 15.5)	16.7 (16.5, 17.0)	10.7 ^†^ (9.9, 11.4)	14.5 ^†^ (13.1, 15.9)	12.2 ^†^ (9.9, 14.9)	15.6 (15.4, 15.9)
≥$75,000	26.5 ^†^ (25.5, 27.5)	44.5 (44.2, 44.9)	18.3 ^†^ (17.4, 19.2)	51.9 ^†^ (50.0, 53.8)	24.9 ^†^ (21.6, 28.5)	39.5 (39.2, 39.8)
Employment status						
Not employed	41.0 (40.0, 42.1)	41.9 (41.5, 42.2)	38.0 ^†^ (36.9, 39.2)	34.2 ^†^ (32.4, 35.9)	49.6 ^†^ (45.9, 53.2)	41.0 (40.7, 41.3)
Employed	59.0 (57.9, 60.0)	58.1 (57.8, 58.5)	62.0 ^†^ (60.8, 63.1)	65.8 ^†^ (64.1, 67.6)	50.4 ^†^ (46.8, 54.1)	59.0 (58.7, 59.3)
Census Region						
Northeast	17.3 ^†^ (16.5, 18.1)	28.3 (28.0, 28.5)	24.1 ^†^ (23.2, 25.0)	31.9 ^†^ (30.4, 33.4)	17.4 ^†^ (14.7, 20.4)	26.6 (26.4, 26.8)
Midwest	14.2 ^†^ (13.6, 14.9)	25.5 (25.3, 25.7)	11.5 ^†^ (10.8, 12.2)	12.0 ^†^ (11.0, 13.1)	18.0 ^†^ (15.7, 20.6)	21.7 (21.5, 21.9)
South	62.6 ^†^ (61.6, 63.6)	34.2 (33.9, 34.5)	41.1 ^†^ (39.9, 42.2)	31.8 ^†^ (30.2, 33.4)	44.6 ^†^ (40.9, 48.3)	38.3 (38.0, 38.6)
West	5.9 ^†^ (5.4, 6.4)	12.0 (11.8, 12.2)	23.4 ^†^ (22.5, 24.3)	24.3 ^†^ (22.5, 26.1)	20.0 ^†^ (17.3, 23.0)	13.4 (13.2, 13.6)

Abbreviations: CI = Confidence Interval. NHOPI = Native Hawaiian or other Pacific Islander. AIAN = American Indian/Alaska Native. * Adults were defined as persons aged ≥18 years. ^†^ *t*-test *p* < 0.05 for significant difference between non-Hispanic white respondents and respondents in another racial/ethnic category. Estimates not reported due to relative standard error >30%.

**Table 6:** Sociodemographic characteristics of large Central metropolitan dwelling adults * by race and ethnicity: Behavioral Risk Factor Surveillance System, United States, 2012–2015.

Sociodemographic Characteristic	Black, Non-Hispanic % (95% CI)	White, Non-Hispanic % (95% CI)	Hispanic % (95% CI)	Asian or NHOPI, Non-Hispanic % (95% CI)	AIAN, Non-Hispanic % (95% CI)	Total
Age (years)						
18–44	50.1 ^†^ (49.3, 51.0)	42.3 (41.9, 42.8)	62.3 ^†^ (61.5, 63.0)	63.6 ^†^ (62.0, 65.1)	52.4 ^†^ (48.7, 56.1)	50.7 (50.4, 51.1)
45–64	34.5 (33.8, 35.3)	35.5 (35.1, 35.9)	28.6 ^†^ (27.9, 29.4)	26.4 ^†^ (25.0, 27.8)	34.4 ^†^ (31.0, 37.9)	32.8 (32.4, 33.1)
65+	15.3 ^†^ (14.8, 15.9)	22.1 (21.9, 22.4)	9.1 ^†^ (8.7, 9.5)	10.0 ^†^ (9.1, 11.1)	13.2 ^†^ (11.1, 15.7)	16.5 (16.3, 16.7)
Sex						
Male	44.8 ^†^ (43.9, 45.6)	49.1 (48.7, 49.5)	49.5 (48.7, 50.4)	49.5 (48.0, 51.0)	49.7 (45.9, 53.5)	48.5 (48.2, 48.9)
Female	55.2 ^†^ (54.4, 56.1)	50.9 (50.5, 51.3)	50.5 (49.6, 51.3)	50.5 (49.0, 52.0)	50.3 (46.5, 54.1)	51.5 (51.1, 51.8)
Marital status						
Not married	71.3 ^†^ (70.5, 72.0)	48.4 (41.0, 41.7)	56.8 ^†^ (56.0, 57.6)	46.8 ^†^ (45.2, 48.3)	64.1 ^†^ (60.4, 67.6)	54.3 (54.0, 54.7)
Married	28.7 ^†^ (28.0, 29.5)	51.6 (51.2, 52.0)	43.2 ^†^ (42.4, 44.0)	53.2 ^†^ (51.7, 54.8)	35.9 ^†^ (32.4, 39.6)	45.7 (45.3, 46.0)
Educational attainment						
<High School	15.9 ^†^ (15.2, 16.6)	5.9 (5.7, 6.2)	40.4 ^†^ (39.6, 41.3)	4.8 ^†^ (4.1, 5.7)	18.2 ^†^ (15.2, 21.6)	16.4 (16.0, 16.7)
High School diploma/GED	31.0 ^†^ (30.2, 31.7)	22.5 (22.1, 22.8)	25.4 ^†^ (24.7, 26.1)	17.0 ^†^ (15.8, 18.3)	28.7 ^†^ (25.5, 32.2)	24.2 (23.9, 24.5)
Some college	34.0 ^†^ (33.2, 34.8)	32.4 (32.0, 32.8)	22.7 ^†^ (22.0, 23.4)	25.7 ^†^ (24.2, 27.2)	34.4 (30.9, 38.2)	29.6 (29.3, 29.9)
College graduate	19.1 ^†^ (18.6, 19.7)	39.2 (38.9, 39.6)	11.5 ^†^ (11.1, 11.9)	52.5 ^†^ (50.9, 54.1)	18.7 ^†^ (16.3, 21.4)	29.9 (29.6, 30.2)
Annual household income						
<$25,000	45.3 ^†^ (44.4, 46.2)	19.1 (18.7, 19.4)	52.8 ^†^ (51.9, 53.7)	23.4 ^†^ (22.0, 24.9)	46.7 ^†^ (42.6, 50.8)	32.6 (32.2, 32.9)
$25,000–$49,999	26.5 ^†^ (25.7, 27.3)	21.4 (21.1, 21.8)	24.9 ^†^ (24.2, 25.7)	19.9 ^†^ (18.6, 21.3)	22.0 (19.0, 25.3)	23.0 (22.7, 23.3)
$50,000–$74,999	11.9 ^†^ (11.3, 12.5)	16.5 (16.2, 16.8)	9.4 ^†^ (8.8, 9.9)	14.1 ^†^ (13.0, 15.2)	10.3 ^†^ (8.3, 12.8)	13.7 (13.4, 13.9)
≥$75,000	16.3 ^†^ (15.6, 17.0)	43.0 (42.6, 43.4)	12.9 ^†^ (12.4, 13.5)	42.6 (41.0, 44.2)	21.0 ^†^ (17.8, 24.7)	30.7 (30.4, 31.1)
Employment status						
Not employed	47.8 ^†^ (47.0, 48.7)	41.7 (41.3, 42.1)	41.7 (40.9, 42.5)	38.5 ^†^ (37.0, 40.1)	48.3 ^†^ (44.5, 52.1)	42.5 (42.1, 42.8)
Employed	52.2 ^†^ (51.3, 53.0)	58.3 (57.9, 58.7)	58.3 (57.5, 59.1)	61.5 ^†^ (59.9, 63.0)	51.7 ^†^ (47.9, 55.5)	57.5 (57.2, 57.9)
Census Region						
Northeast	23.4 ^†^ (22.8, 24.1)	17.4 (17.1, 17.6)	15.5 ^†^ (15.1, 16.0)	19.8 ^†^ (18.7, 21.0)	15.4 (13.1, 18.1)	18.1 (17.9, 18.3)
Midwest	23.1 ^†^ (22.5, 23.8)	19.6 (19.4, 19.9)	7.3 ^†^ (6.9, 7.7)	7.1 ^†^ (6.6, 7.6)	17.4 (14.8, 20.3)	15.9 (15.7, 16.1)
South	39.4 ^†^ (38.6, 40.2)	27.9 (27.5, 28.2)	32.1 ^†^ (31.3, 32.8)	13.8 ^†^ (12.9, 14.8)	30.5 (27.0, 34.2)	29.6 (29.3, 29.9)
West	14.0 ^†^ (13.4, 14.7)	35.1 (34.7, 35.4)	45.1 ^†^ (44.4, 45.9)	59.2 ^†^ (57.8, 60.7)	36.7 (33.2, 40.4)	36.4 (36.1, 36.6)

Abbreviations: CI = Confidence Interval. NHOPI = Native Hawaiian or other Pacific Islander. AIAN = American Indian/Alaska Native. * Adults were defined as persons aged ≥18 years. ^†^ *t*-test *p* < 0.05 for significant difference between non-Hispanic white respondents and respondents in another racial/ethnic category. Estimates not reported due to relative standard error >30%.

**Table 7:** Age adjusted mortality rates per 100,000 population for the five leading causes of death by race/ethnicity * groups and Urbanization—National Vital Statistics System, United States, 2012–2015.

2013 Urbanization	White	Black	AIAN	Hispanic	API
All causes					
Large Central Metro	705.8	887.5	608.7	525.4	393.4
Large Fringe Metro	706.7	771.1	542.4	456.9	354.7
Medium Metro	753.1	917.8	747.5	578.3	464.3
Small Metro	777.8	940.6	804.7	538.2	423.1
Micropolitan (non-metro)	826.5	988.7	909.8	601.3	488.1
NonCore (non-metro)	842.9	968.5	1033.3	570.5	377
U.S.	748.1	879.1	793.8	529.1	399.4
Heart disease					
Large Central Metro	167.2	222	123.3	124.6	89.1
Large Fringe Metro	161.5	180.3	115.2	97.3	77.2
Medium Metro	167.6	211.6	151.2	120.2	101.1
Small Metro	175.1	223.9	147	108.9	94.4
Micropolitan (non-metro)	190.9	234	172.9	129.5	118.5
NonCore (non-metro)	198.4	247.5	193.3	126.1	82.2
U.S.	171.7	213	154.1	118.6	89.2
Malignant neoplasms					
Large Central Metro	162.1	196.9	114.6	115.9	104.1
Large Fringe Metro	162.6	172	104.5	101.6	88.5
Medium Metro	166.5	195.6	141.1	117	109
Small Metro	169.5	195.4	128.5	110.2	101
Micropolitan (non-metro)	178	208.2	152.3	114	108.6
NonCore (non-metro)	180.1	201	181.2	107.4	86
U.S.	167.1	191.8	140.8	113	101
Chronic lower respiratory disease					
Large Central Metro	40.8	30.4	30.1	18	13.7
Large Fringe Metro	41.2	24.1	35.9	15.3	10.5
Medium Metro	47.3	31.8	40.9	18.7	12.9
Small Metro	50.4	32.3	34.2	20.3	12.4
Micropolitan (non-metro)	56	34.1	41.3	20.9	12.5
NonCore (non-metro)	56.7	31.1	48.4	20.5	11.4
U.S.	46.5	29.6	39.3	18	12.8
Cerebrovascular disease					
Large Central Metro	32.5	48.1	25.5	29.9	29.3
Large Fringe Metro	33.5	47.3	23.6	28.7	26.4
Medium Metro	36	54.7	32.7	33.1	33.7
Small Metro	37.5	58.4	30.4	30.9	30.8
Micropolitan (non-metro)	40.9	61.3	33.8	32.8	40.2
NonCore (non-metro)	40.6	59.4	40.2	30.5	29.1
U.S.	35.7	50.9	31.8	30.5	29.7
Unintentional injury					
Large Central Metro	40.5	35.3	51.2	24.6	13.9
Large Fringe Metro	42	28.2	37.5	23.6	14.3
Medium Metro	47.2	37.4	61.8	30.7	20.1
Small Metro	46.9	36.3	87.4	33.7	19
Micropolitan (non-metro)	53.1	42.5	90.4	38.8	22.5
NonCore (non-metro)	60.8	45.4	107	43.9	21
U.S.	45.8	34.9	74.3	27.2	15.3

Abbreviations: AI/AN = American Indian/Alaska Native; API = Asian and Pacific Islander; * All races are non-Hispanic.

**Table 8:** Disparities in age adjusted mortality rates for race/ethnicity * groups compared to non-Hispanic white population by urbanization—National Vital Statistics System, United States, 2012–2015.

2013 Urbanization	Black	AIAN	Hispanic	API
All causes				
Large Central Metro	181.7	−97.1	−180.4	−312.4
Large Fringe Metro	64.4	−164.3	−249.8	−352
Medium Metro	164.7	−5.6 ^†^	−174.8	−288.8
Small Metro	162.8	26.9	−239.6	−354.7
Micropolitan (non-metro)	162.2	83.3	−225.2	−338.4
NonCore (non-metro)	125.6	190.4	−272.4	−465.9
U.S.	131	45.7	−219	−348.7
Heart disease				
Large Central Metro	54.8	−43.9	−42.6	−78.1
Large Fringe Metro	18.8	−46.3	−64.2	−84.3
Medium Metro	44	−16.4	−47.4	−66.5
Small Metro	48.8	−28.1	−66.2	−80.7
Micropolitan (non-metro)	43.1	−18	−61.4	−72.4
NonCore (non-metro)	49.1	−5.1 ^†^	−72.3	−116.2
U.S.	41.3	−17.6	−53.1	−82.5
Malignant neoplasms				
Large Central Metro	34.8	−47.5	−46.2	−58
Large Fringe Metro	9.4	−58.1	−61	−74.1
Medium Metro	29.1	−25.4	−49.5	−57.5
Small Metro	25.9	−41	−59.3	−68.5
Micropolitan (non-metro)	30.2	−25.7	−64	−69.4
NonCore (non-metro)	20.9	1.1 ^†^	−72.7	−94.1
U.S.	24.7	−26.3	−54.1	−66.1
Chronic lower respiratory disease				
Large Central Metro	−10.4	−10.7	−22.8	−27.1
Large Fringe Metro	−17.1	−5.3	−25.9	−30.7
Medium Metro	−15.5	−6.4	−28.6	−34.4
Small Metro	−18.1	−16.2	−30.1	−38
Micropolitan (non-metro)	−21.9	−14.7	−35.1	−43.5
NonCore (non-metro)	−25.6	−8.3	−36.2	−45.3
U.S.	−16.9	−7.2	−28.5	−27.1
Cerebrovascular disease				
Large Central Metro	15.6	−7	−2.6	−3.2
Large Fringe Metro	13.8	−9.9	−4.8	−7.1
Medium Metro	18.7	−3.3	−2.9	−2.3
Small Metro	20.9	−7.1	−6.6	−6.7
Micropolitan (non-metro)	20.4	−7.1	−8.1	−0.7 ^†^
NonCore (non-metro)	18.8	−0.4 ^†^	−10.1	−11.5
U.S.	15.2	−3.9	−5.2	−6
Unintentional injury				
Large Central Metro	−5.2	10.7	−15.9	−26.6
Large Fringe Metro	−13.8	−4.5	−18.4	−27.7
Medium Metro	−9.8	14.6	−16.5	−27.1
Small Metro	−10.6	40.5	−13.2	−27.9
Micropolitan (non-metro)	−10.6	37.3	−14.3	−30.6
NonCore (non-metro)	−15.4	46.2	−16.9	−39.8
U.S.	−10.9	28.5	−18.6	−30.5

Abbreviations: AI/AN = American Indian/Alaska Native; API = Asian and Pacific Islander; * All races are non-Hispanic; ^†^ Not significantly different from non-Hispanic white; other disparities are statistically significant (*p* < 0.05, *z*-test).

**Table 9:** Contribution of the six rural-urban areas to age-specific disparities in all-cause mortality rates per 100,000 population between NHB (NHB) and NHW (NHW) populations in the U.S.—National Vital Statistics System, United States, 2012–2015.

	NHB Mortality Rate (MR)	NHW MR	NHB vs. NHW Disparity (NHW MR-NHW MR)	NHB Population Ratio	NHW Population Ratio	Contribution to National NHB vs. NHW Disparity
Age 20–44						
Large Central Metro (R1)	200.1	111.8	88.3	0.42	0.25	56.61
Large Fringe Metro (R2)	155.8	128.7	27.1	0.23	0.25	3.48
Medium Metro (R3)	196.9	140.6	56.3	0.18	0.22	5.06
Small Metro (R4)	186.9	140.3	46.6	0.07	0.11	−2.33
Micropolitan (non-metro) (R5)	203.4	161.7	41.7	0.05	0.10	−5.56
NonCore (non-metro) (R6)	210.9	186.1	24.8	0.04	0.07	−4.00
U.S.	188.89	135.63	53.26			53.26
Age 45–64						
Large Central Metro (R1)	949.0	591.3	357.7	0.42	0.22	274.92
Large Fringe Metro (R2)	692.9	540.1	152.8	0.24	0.28	15.45
Medium Metro (R3)	947.8	661.3	286.5	0.17	0.22	21.39
Small Metro (R4)	1005.4	702.7	302.7	0.07	0.10	−8.44
Micropolitan (non-metro) (R5)	1073.5	748	325.5	0.05	0.11	−21.67
NonCore (non-metro) (R6)	1082.1	785.7	296.4	0.04	0.08	−13.42
U.S.	904.0	635.8	268.2			268.22
Age ≥ 65						
Large Central Metro (R1)	4381.7	4439.9	−58.2	0.44	0.21	1000.51
Large Fringe Metro (R2)	3835.2	4301.6	−466.4	0.21	0.25	−267.42
Medium Metro (R3)	4472.4	4385.1	87.3	0.17	0.22	−211.73
Small Metro (R4)	4624.1	4454.9	169.2	0.07	0.11	−200.26
Micropolitan (non-metro) (R5)	4934.2	4660.8	273.4	0.06	0.11	−242.14
NonCore (non-metro) (R6)	4858.1	4618.7	239.4	0.05	0.09	−161.16
U.S.	4353.1	4435.3	−82.2			−82.2

## References

[1] Cossman J., James W., Wolf J.K. (2017). The differential effects of rural health care access on race-specific mortality. SSM Popul. Health.

[2] Eberhardt M.S., Pamuk E.R. (2004). The importance of place of residence: Examining health in rural and nonrural areas. Am. J. Public Health.

[3] Fitzpatrick K., LaGory M. (2002). Unhealthy Places: The Ecology of Risk in the Urban Landscape.

[4] Fitzpatrick K.M., LaGory M. (2003). “Placing” health in an urban sociology: Cities as mosaics of risk and protection. City Community.

[5] Bernard P., Charafeddine R., Frohlich K.L., Daniel M., Kestens Y., Potvin L. (2007). Health inequalities and place: A theoretical conception of neighbourhood. Soc. Sci. Med..

[6] White K., Haas J.S., Williams D.R. (2012). Elucidating the role of place in health care disparities: The example of racial/ethnic residential segregation. Health Serv. Res..

[7] Morenoff J.D., Lynch J.W. (2004). What makes a place healthy? Neighborhood influences on racial/ethnic disparities in health over the life course. Critical Perspectives on Racial and Ethnic Differences in Health in Late Life.

[8] Moy E., Garcia M.C., Bastian B., Rossen L.M., Ingram D.D., Faul M., Massetti G.M., Thomas C.C., Hong Y., Yoon P.W. (2017). Leading causes of death in nonmetropolitan and metropolitan areas—United states, 1999–2014. MMWR Surveill. Summ..

[9] Ivey-Stephenson A.Z., Crosby A.E., Jack S.P.D., Haileyesus T., Kresnow-Sedacca M.J. (2017). Suicide trends among and within urbanization levels by sex, race/ethnicity, age group, and mechanism of death-united states, 2001–2015. MMWR Surveill. Summ..

[10] Mack K.A., Clapperton A.J., Macpherson A., Sleet D., Newton D., Murdoch J., Mackay J.M., Berecki-Gisolf J., Wilkins N., Marr A. (2017). Trends in the leading causes of injury mortality, australia, canada and the united states, 2000–2014. Can. J. Public Health.

[11] Case A., Deaton A. (2015). Rising morbidity and mortality in midlife among white non-hispanic americans in the 21st century. Proc. Natl. Acad. Sci. USA.

[12] Lichter D.T., McLaughlin D.K. (1995). Changing economic opportunities, family structure, and poverty in rural areas. Rural Sociol..

[13] Singh G.K., Siahpush M. (2014). Widening rural-urban disparities in all-cause mortality and mortality from major causes of death in the USA, 1969–2009. Urban Health.

[14] James W., Cossman J.S. (2017). Long-term trends in black and white mortality in the rural united states: Evidence of a race-specific rural mortality penalty. J. Rural Health.

[15] Harris J.K., Beatty K., Leider J.P., Knudson A., Anderson B.L., Meit M. (2016). The double disparity facing rural local health departments. Annu. Rev. Public Health.

[16] MacKinney A., Coburn A., Lundblad J., McBride T., Mueller K., Watson S. (2014). Access to Rural Health Care—A Literature Review and New Synthesis.

[17] Meit M., Knudson A., Gilbert T., Yu A.T.-C., Tanenbaum E., Ormson E., Popat M. (2014). The 2014 Update of the Rural-Urban Chartbook.

[18] Strine T.W., Beck L.F., Bolen J., Okoro C., Dhingra S., Balluz L. (2010). Geographic and sociodemographic variation in self-reported seat belt use in the united states. Accid. Anal. Prev..

[19] Ingram D.D., Franco S.J. (2014). 2013 nchs urban-rural classification scheme for counties. Vital and Health Statistics, Series 2: Data Evaluation and Methods Research.

[20] Centers for Disease Control and Prevention Behavioral Risk Factor Surveillance System: Survey Data & Documentation. https://www.cdc.gov/brfss/data_documentation/index.htm.

[21] U.S. Census Bureau (2000). Census Regions and Divisions of the United States.

[22] CDC WONDER. https://wonder.cdc.gov.

[23] Kulshreshtha A., Goyal A., Dabhadkar K., Veledar E., Vaccarino V. (2014). Urban-rural differences in coronary heart disease mortality in the united states: 1999–2009. Public Health Rep..

[24] Barnett E., Halverson J. (2000). Disparities in premature coronary heart disease mortality by region and urbanicity among black and white adults ages 35–64, 1985–1995. Public Health Rep..

[25] National Advisory Committee on Rural Health and Human Services (2015). Mortality and Life Expectancy in Rural America: Connecting the Health and Human Service Safety Nets to Improve Health Outcomes over the Life Course.

[26] Garcia M.C., Faul M., Massetti G., Thomas C.C., Hong Y., Bauer U.E., Iademarco M.F. (2017). Reducing potentially excess deaths from the five leading causes of death in the rural united states. MMWR Surveill. Summ..

[27] Anderson T.J., Saman D.M., Lipsky M.S., Lutfiyya M.N. (2015). A cross-sectional study on health differences between rural and non-rural u.S. Counties using the county health rankings. BMC Health Serv. Res..

[28] Blake K.D., Moss J.L., Gaysynsky A., Srinivasan S., Croyle R.T. (2017). Making the case for investment in rural cancer control: An analysis of rural cancer incidence, mortality, and funding trends. Cancer Epidemiol. Prev. Biomark..

[29] Fairchild H.H., Tucker M.B. (1982). Black residential mobility: Trends and characteristics. J. Soc. Issues.

[30] Black D.A., Sanders S.G., Taylor E.J., Taylor L.J. (2015). The impact of the great migration on mortality of african americans: Evidence from the deep south. Am. Econ. Rev..

[31] Charles C.Z. (2003). The dynamics of racial residential segregation. Annu. Rev. Sociol..

[32] Cotter D.A. (2002). Poor people in poor places: Local opportunity structures and household poverty. Rural Sociol..

[33] Albrecht D.E., Albrecht C.M., Murguia E. (2005). Minority concentration, disadvantage, and inequality in the nonmetropolitan united states. Sociol. Q..

[34] Caldwell J.T., Ford C.L., Wallace S.P., Wang M.C., Takahashi L.M. (2016). Intersection of living in a rural versus urban area and race/ethnicity in explaining access to health care in the united states. Am. J. Public Health.

[35] Probst J.C., Moore C.G., Glover S.H., Samuels M.E. (2004). Person and place: The compounding effects of race/ethnicity and rurality on health. Am. J. Public Health.

[36] Office of Management and Budget (OMB) Revisions to the Standards for the Classification of Federal Data on Race and Ethnicity. https://obamawhitehouse.archives.gov/omb/fedreg_1997standards.

[37] Arias E., Heron M., Hakes J.K. (2016). The validity of race and hispanic-origin reporting on death certificates in the united states: An update. Vital and Health Statistics, Series 2: Data Evaluation and Methods Research.

[38] Arias E., Schauman W.S., Eschbach K., Sorlie P.D., Backlund E. (2008). The validity of race and hispanic origin reporting on death certificates in the united states. Vital and Health Statistics. Series 2, Data Evaluation and Methods Research.

